# Mixed Feelings of Children and Adolescents with Unilateral Congenital Below Elbow Deficiency: An Online Focus Group Study

**DOI:** 10.1371/journal.pone.0037099

**Published:** 2012-06-08

**Authors:** Ingrid G.M. de Jong, Heleen A. Reinders-Messelink, Wim G.M. Janssen, Margriet J. Poelma, Iris van Wijk, Corry K. van der Sluis

**Affiliations:** 1 Department of Rehabilitation Medicine, University Medical Center Groningen, Groningen, The Netherlands; 2 Department of Rehabilitation Medicine, Erasmus MC, Rotterdam, The Netherlands; 3 Department of Rehabilitation Medicine, Rehabilitation Centre De Sint Maartenskliniek, Nijmegen, The Netherlands; 4 Department of Rehabilitation Medicine, Rehabilitation Centre De Hoogstraat, Utrecht, The Netherlands; Weill Cornell Medical College, United States of America

## Abstract

The existing literature is inconsistent about the psychosocial functioning of children and adolescents with Unilateral Congenital Below Elbow Deficiency (UCBED). The objective of this qualitative study was to explore the psychosocial functioning of children and adolescents with UCBED in terms of their feelings about the deficiency and what helps them to cope with those feelings. Additionally, the perspectives of prosthesis wearers and non-wearers were compared, as were the perspectives of children, adolescents, parents and health professionals. Online focus group interviews were carried out with 42 children and adolescents (aged 8–12, 13–16 and 17–20), 16 parents and 19 health professionals. Questions were asked about psychosocial functioning, activities, participation, prosthetic use or non-use, and rehabilitation care. This study concerned remarks about psychosocial functioning. Children and adolescents with UCBED had mixed feelings about their deficiency. Both negative and positive feelings were often felt simultaneously and mainly depended on the way people in the children’s environment reacted to the deficiency. People staring affected the children negatively, while support from others helped them to cope with the deficiency. Wearing a prosthesis and peer-to-peer contact were also helpful. Non-wearers tended to be more resilient than prosthesis wearers. Wearers wore their prosthesis for cosmetic reasons and to prevent them from negative reactions from the environment. We recommend that rehabilitation teams make parents aware of their great influence on the psychosocial functioning of their child with UCBED, to adjust or extend the currently available psychosocial help, and to encourage peer-to-peer contact.

## Introduction

Children and adolescents with Unilateral Congenital Below Elbow Deficiency (UCBED) have a visible limb difference. As such, they may be at risk of psychological adjustment problems [1], [2]. However, the literature is inconsistent when it comes to psychological consequences of UCBED, since previously it had been revealed that children with UCBED who were fitted with myoelectric prosthetic hands were as mentally healthy as their able-bodied peers [3].

The psychosocial consequences of UCBED are mostly described from the parents’ perspective. Parents stated that among the challenges of raising children with UCBED were managing grief-related emotions and concerns about their children, making medical decisions, and finding an appropriate way to communicate with their children [4]. Parents also revealed that difficulties were solved by their children’s strong personality, their connections with other families with children with similar deficiencies, emotional support from friends or family, and humour [4]. However, it remains unclear how children themselves feel about their psychosocial functioning. In their study, Ylimäinen et al. [5] found that parents tend to overemphasise the problems caused by the deficiency compared to how children rated their own health-related quality of life [5]. Hence, they underlined the importance of taking the children’s own ratings into consideration in addition to the parents’ ratings [5].

This paper presents a qualitative study in which children and adolescents with UCBED shared their experiences of being different to their peers and discussed what helped them handle the feelings caused by their deficiency. To be able to compare the results with what is known from the literature, parents of children with UCBED were also included in this study. Furthermore, to permit us to compare the perspectives of several groups, health professionals participated. Such a comparison might have consequences for clinical practice, if for example professionals have a different view on the psychosocial functioning of children with UCBED compared to the children or adolescents themselves, or their parents.

The aims of this study were to explore the psychosocial functioning of children and adolescents with UCBED in terms of feelings related to their deficiency and what helps them learn to cope with those feelings. Secondly, we compared the perspectives of prosthesis wearers and non-wearers regarding feelings and coping strategies, and the perspectives of the children, adolescents, parents, and health professionals.

## Methods

### Study Design

Online focus group interviews were selected as an appropriate procedure to gather people’s opinions and experiences for this study [6], [7]. Between 8 and 15 participants per group were recommended for online focus group interviews [8]–[12].

### Ethics Statement

This study was approved by the Medical Ethical Committee of the University Medical Center Groningen, the Netherlands. Written informed consent was received from all participants who took part in the study. In case of minors, the parents or guardians gave informed consent.

### Participants

Five groups of participants were included in the study: three groups of children and adolescents with UCBED aged 8 to 20, parents and health professionals. Children and adolescents were divided into the following age groups: 8–12, 13–16 and 17–20 years. These groups matched primary school age, secondary school age, and the age at which an adolescent generally starts to follow a secondary or higher education.

The participants (except for health professionals) were recruited from four Dutch rehabilitation centres and patient organisations. Professionals were solicited via several rehabilitation institutions and orthopaedic workshops in the Netherlands. We randomly selected a total of 25 participants per group (taking age, gender, referring centre and prosthetic use or non-use into account).

### Procedure

A website was designed to facilitate the online focus group interviews. It was equipped with a separate forum for each group of participants. Over seven consecutive days (time span interviews) participants logged into their forum at a time and place that was most suitable for them. This is known as the asynchronous form of online focus group interviews [6]. Every day a new discussion topic was placed online, with the exception of the last two days, during which participants were given the opportunity to bring in their own topics. On day 3 we asked the participants about psychosocial functioning: ‘Tell us how you feel about being different from other children because of your arm?’ (this was the formulation used for the youngest children). In addition to psychosocial functioning – the topic of the present study – topics included activities (day 1), participation (day 2), prosthetic use or non-use (day 4), and rehabilitation care (day 5). Parents and professionals were asked to formulate their reactions from the child’s perspective, which enabled the comparison of group perspectives. During the study week, two authors (IdJ and HRM) were active as moderators on the forums. Their role was to provoke discussion by asking additional questions, to check if participants complied with the rules, and to send e-mails to non-respondents. Both moderators were researchers in the field of child and hand rehabilitation. During the interviews, the moderators had an extensive contact with a rehabilitation physician with great experience in working with this particular group of patients.

### Data Analysis

All remarks on psychosocial functioning which had been made during the whole week were taken into consideration for data analysis. An inductive and deductive framework approach was applied to analyse the data from the online focus group interviews [13]. The framework included subjects frequently mentioned by participants during the study week, clustered into themes and related to the research questions. These themes could be further divided into subthemes [13]. For example, a theme of this study’s framework was ‘feelings’, which was divided into subthemes such as ‘acceptance’, ‘mixed feelings’, ‘shame’ and ‘anger’. Before coding the entire dataset, two of the authors (IdJ and HRM) both analysed ten percent of the data using the thematic framework and reviewed their results, after which the final thematic framework was composed. The data were coded using this final framework by one author (IdJ), who went through the data for all five topics and collected all the quotes on psychosocial functioning into a matrix. The matrix was organised so that each row contained the remarks from one participant and each column comprised the remarks from all participants on a given subtheme. To extract the most important results, three authors (IdJ, HRM, CvdS) went through the whole matrix individually and discussed their results. After that, an additional analysis was performed on data that was sorted for prosthesis wearers and non-wearers.

## Results

Seventy-seven children, adolescents, parents and health professionals out of a total of 125 eligible participants (62%) participated in the online focus group interviews. No differences between participants and non-participants were found regarding age, gender and referring centre. The response rate per group varied from 48 to 76 percent (Table 1). Among the participating professionals were rehabilitation physicians, prosthetists, occupational and physical therapists, and psychologists. 884 of all quotes concerned remarks about psychosocial functioning (Table 1). There was no difference in the number of subjects who made remarks about the psychosocial topic and the other topics.

**Table 1:** Characteristics of participants of online focus groups.

Group	Participants	Gender	Distribution	Age	Wearers	Quotes
	*N* (response rate %)^a^	M, F	*N* b	Mean [SD]	*N* (%)	*N* c
**8–12 y**	25, 17, 17 (68)	9, 8	3, 3, 4, 4, 3	9.9 [1.3]	2 (12)	149
**13–16 y**	25, 15, 13 (52)	3,10	2, 3, 3, 5, 0	14.9 [1.4]	6 (46)	225
**17–20 y**	25, 13, 12 (48)	4, 8	2, 3, 4, 3, 0	18.3 [1.1]	5 (42)	109
**Parents**	25, 19, 16 (64)	10, 6^d^	3, 3, 4, 6, 3^d^	12.7 [3.8]^d^	1 (6)^d^	246
**Professionals**	25, 19, 19 (76)	8, 11	5, 4, 3, 5, 2	−	−	155
**Total**	125, 83, 77 (62)	−	15, 16, 18, 23, 8	13.9 [3.8]^e^	13 (31)^e^	884

a Number of subjects eligible to recruit, recruited, participated in study and response rate (%).

b Number of participants across each of the 4 cooperating centres; the last number reflects the number of participants recruited through other centres/organisations.

c Number of quotes concerning psychosocial functioning.

d Characteristics of the children of participating parents.

e Based on the characteristics of the three children/adolescents groups.

### 1 Children and Adolescents

#### 1.1 Feelings

Few of the children and adolescents had exclusively positive or negative feelings towards their deficiency, most of them reported both. The youngest children (aged 8–12) were particularly likely to describe mixed feelings about their arm:

“Sometimes it’s fun to have a short arm, but sometimes it isn’t, because sometimes I’m ashamed of my short arm.”

(11-year-old girl, non-wearer)

“I don’t mind [having one hand]… But I’d prefer to have a normal hand.”

(8-year-old girl, wearer)

Mixed feelings about the deficiency were, for example, encountered in friendships and relationships. Making contact and starting up new friendships with peers appeared to be no problem for the youngest children. Several adolescents, on the other hand, described difficulties with making contact and starting up relationships. These difficulties were often caused by insecurity about what others would think about the deficiency.

Negative feelings reported by children and adolescents were shame, feelings of being different than peers, being fed up with the deficiency and wishing to be more like everybody else. These negative feelings were generally caused by the negative reactions which children and adolescents got from people in their environment. What children and adolescents found by far the most aggravating was that people, especially strangers and other children, constantly stared at the short arm:

“It’s really annoying that people stare at it [the short arm] continuously.”

(16-year-old girl, wearer)

“When I’m walking in town, they [strangers] look at you as if you’re a whole other person, and then I get ashamed of myself.”

(13-year-old girl, non-wearer)

Other reactions from the environment which affected children and adolescents with UCBED negatively were teasing (which was especially common in primary school), rejection, being treated differently than peers, and people being scared of the short arm. Negative feelings about the short arm were also described in situations where a child had to meet new people (transition to a new school, going out) or in which the short arm became evident (wearing a t-shirt during summer or vacation). Children aged 13 to 16 were particularly prone to having negative feelings related to UCBED, reporting feelings of shame and being different than peers. Some children and adolescents aged 13 to 20 described puberty as a tough time, since appearance became more important then. This caused insecurity about the short arm, which in most cases disappeared after puberty. Despite the negative feelings often experienced during puberty, several children of 13 years and older stated that they no longer wished for a sound hand:

“I was born this way and even if it would be possible to “get” another arm, I wouldn’t want that.”

(16-year-old girl, non-wearer)

The remarks of children in the youngest age group (aged 8–12) were in contrast to the former statement. This group of children preferred to have two sound arms.

Positive feelings towards the short arm included pride, acceptance, satisfaction, being okay with being different than peers, and not feeling different at all. These kinds of feelings were usually reported by adolescents (aged 17–20); they were no longer ashamed of their arm, and they described that feelings of acceptance dominated. Positive feelings towards the deficiency were also to a great extent determined by the way people in the child’s environment reacted to the deficiency. Several children and adolescents described receiving positive reactions from their environment, such as acceptance, respect and admiration for the way they functioned with their deficiency. Adolescents gave more examples of these positive reactions from the environment than the two younger age groups.

#### 1.2 What helps?

Support from people in the direct environment of the child (family, friends and classmates) was very important and helped children and adolescents to cope with their short arm (Table 2). This was expressed particularly frequently by children up to the age of 16. Wearing a prosthesis also seemed helpful for several children and adolescents. A prosthesis was often chosen for cosmetic reasons, to prevent staring and other negative reactions from the environment:

“The reason for me to start wearing a prosthesis was that I was annoyed by the constant staring of people. People happen to remember the first impression they have of someone. It’s not that I’m ashamed of it, but I just don’t want to be seen as the boy with one arm.”

(20-year-old boy, wearer)

**Table 2:** Things that help children/adolescents with UCBED to cope with the deficiency.

	8–12 y	13–16 y	17–20 y	Parents	Professionals
Wearing a prosthesis^a^	+	+	+	+	+
Contact with fellow sufferers	−	+	−	+	+
Support from the rehabilitation team	−	+	−	+	+
Humour	+	−	−	−	−
Support from people in the environment	+	+	+/−	+/−	+
Hiding the short arm	+/−	+	+/−	+	−
The children’s persistence, self-confidence	−	−	+	+	+
Parental openness towards their child	−	−	−	+	+/−

(+): frequently mentioned by participants; (+/−): mentioned once; (−): not mentioned.

a Reported by both prosthesis wearers and non-wearers.

Contact with similar others and help from the rehabilitation team was particularly important for children aged 13–16. For some children, hiding the short arm seemed to be the best option to avoid reactions:

“In primary school, I used to hide my arm in the drawer of my desk. I usually wear long-sleeved shirts, even during gym class. I’m scared of wearing a t-shirt.”

(13-year-old girl, non-wearer)

Furthermore, children and adolescents often choose to provide information on their deficiency in order to put a stop to people from their environment who are staring at them. In contrast, they did not feel the need to talk about the deficiency to parents or psychologists. Finally, some of the children and adolescents felt that people in their environment should accept them as they are.

“It doesn’t matter to me at all what others think. And when they look at me, I’ll look back or I’ll ask: “what is the matter?”. They have to take me as I am.”

(18-year-old girl, wearer)

#### 1.3 Differences between prosthesis wearers and non-wearers

The extent to which children and adolescents had positive, negative or mixed feelings related to the short arm did not differ between prosthesis wearers and non-wearers. A difference was found, however, in the participants’ remarks about their wish to have a sound hand. Some non-wearers indicated that they would rather have a sound hand if they had had the choice, while other non-wearers had stopped wishing for a sound hand because they felt complete without it. The latter remark was not made by prosthesis wearers. Wearers only indicated they would like two sound hands. Furthermore, non-wearers gave more examples of negative reactions from people in their environment – such as rejection, teasing, being treated differently or being stared at – than wearers. Another difference between wearers and non-wearers was that prosthesis wearers indicated more often than non-wearers that they found the summer or going on holidays difficult, because their deficiency would then become more obvious.

Non-wearers were more often of the opinion that others “have to take me as I am” or “if they think I’m weird, they are not worth being my friends”, compared to prosthesis wearers. This seemed a helpful strategy for them to cope with reactions from people in the environment. Generally, non-wearers described more extensively than wearers how they responded to reactions from people in the environment. Non-wearers used humour more often than wearers to deal with reactions. An example:

“A man at the ski-lift once tried to help me, and pulled my glove. There I was already on the lift and he was left holding my glove. His face turned white, because he thought he had pulled off my hand. My whole family laughed. Sometimes, laughing is the best way to deal with it.”

(11-year-old boy, non-wearer)

Furthermore, non-wearers were more willing to explain UCBED to people in their environment, but also more likely to hide their short arm than prosthesis wearers.

### 2 Parents

#### 2.1 Feelings from their children’s perspective

Parents are well aware of the fact that their children’s negative feelings towards their arm arise from the staring of strangers. According to parents, staring was what bothered their children most of all the reactions from people in the environment. A few parents mentioned teasing, rejection and being treated differently as further reactions from the environment which affected their children negatively, but these kinds of reactions were mentioned less often than by the children and adolescents themselves.

Parents described that their children had mainly positive feelings about their deficiency: the children had accepted their deficiency and felt okay with being different than peers. Another difference was that parents gave fewer examples of positive reactions from the environment (such as acceptance, respect and admiration) compared to children and adolescents.

#### 2.2 Parents’ own feelings

Most parents described that they have gone through different stages of acceptance after the birth of their child. Right after birth, negative feelings such as anger, shame and guilt dominated. Sometimes these feelings were quickly replaced by acceptance. With other parents it took longer, because they saw the deficiency as a handicap in their children’s early years. These feelings disappeared when the parents noticed that their children were doing very well and that they developed just like any other child:

““How can you accept your child’s deficiency?” Our acceptance came when she showed us what she was capable of.”

(Parent of a 9-year-old girl, non-wearer)

A lot of parents explained the exact moment when their children came to realise they were different than peers. That moment appeared not to be attached to a certain age, but was triggered by events in the children’s early lives. For example, when children with UCBED got a brother or sister with two hands, or when other people or children said things about the short arm:

“The awareness of the short arm came for my son when he was 3 or 4 years old. He overheard a conversation between another boy and his mother. To the boy’s question of why my son had only one hand, his mother responded that he just had bad luck. Up to that point my son had not thought that he had “bad luck”. However, that moment in the gym was the moment he realised that his hand was not going to grow any further and that no doctor could ever ‘fix’ it.”

(Parent of a 17-year-old boy, non-wearer)

#### 2.3 What helps?

Parents mentioned, just like children and adolescents, that wearing a prosthesis and peer-to-peer contact with similar others can be helpful in learning to live with UCBED (Table 2). In addition, parents also described the value of the assistance from the rehabilitation team. Both peer-to-peer contact and assistance from the rehabilitation team was not only helpful for children, but also for parents. It offered understanding and recognition and reassured parents about their child’s future.

Several parents said that the best way to support their child was to be open about the short arm and to speak about it positively, to give their children the feeling that the arm is nothing to be ashamed of. In addition, parents reported that their children’s personalities were important in coping with the deficiency. Most parents described their children as blessed with a good sense of humour, persistence and self-confidence and they felt that these characteristics helped their children deal with their deficiency.

### 3 Health professionals

#### 3.1 Feelings

Health professionals only gave very few examples of the feelings which children or adolescents with UCBED have towards their deficiency:

“Every child has a moment while growing up when he or she feels fed up with having a short arm; whether that is sorrow…having a hard time responding to other’s reactions…or being tired of explaining that you’re not restricted in activities or participation…”.

#### 3.2 What helps?

Health professionals described that assistance from rehabilitation teams should include psychosocial help and education of children and parents. Furthermore, they recommended that there should be a collaboration between the rehabilitation team and the child’s environment, such as school, family or sports club. Professionals considered psychosocial help of importance in teaching children to talk about the deficiency.

Professionals stressed that the parents’ coping strategy is of great importance in how children learn to deal with the fact that they have a short arm. If parents can accept their children’s deficiency and behave normally about it, it is easier for the children to cope with it. Remarkably, this parental influence was not mentioned by the parents themselves.

## Discussion

The existing literature is inconsistent about the psychosocial functioning of children and adolescents with UCBED. Several studies which investigated factors mediating the relationship between limb deficiencies and psychological functioning [14]–[19] presume children with limb deficiencies to be at risk of psychological problems [1], [2]. Despite the fact that children with chronic physical handicaps have been found to be at risk of psychological adjustment problems [1], [2], there is no evidence for the belief that this relationship also applies to children with limb deficiencies. In fact, in their study, Hermansson et al. [3] showed that children with upper limb deficiency are as well-adjusted psychosocially as their able-bodied peers. Although in our study many psychosocial topics were raised, these did not seem to be severe. As such, our results seem to confirm Hermansson et al.’s conclusions [3].

The results of our study show that children and adolescents had mixed feelings about their deficiency. The environment can be seen as the triggering factor for the psychosocial functioning of a child or adolescent with UCBED. Our results show that psychological adjustment is not only determined by the way the child sees him or herself (perceived physical appearance; [14]), but the environment plays a significant role too. From earlier research into the body image of adults with physical disabilities is known that disability is not biologically determined, but rather socially constructed [20]. The consequences of environmental factors can work in two ways: if a physically disabled person lives in an environment of acceptance, the acceptance of others leads to self-acceptance. In contrast, devaluation by society can have a negative impact on body image [21]. Similar conclusions were drawn by Green [22] for the effects of social stigma on children with disabilities and their families. Social stigma can cause fear of being stigmatised, which in turn can limit a person’s interactions with peers. However, positive experiences with social interactions strengthen children and take away the fear of being stigmatised [22]. Monitoring whether a child with UCBED experiences sufficient positive interactions in daily life is a possible role for the professionals in the rehabilitation team.

Besides support from the environment, wearing a prosthesis and contact with similar others were helpful in coping with a limb deficiency. It had previously been suggested that prosthetic use could promote social acceptance [23], but our study is the first to show that wearing a prosthesis can help children with UCBED to resist negative reactions from their environment. Although prostheses help a limited number of people overcome their limitations in activities and participation [24], their great value to the psychosocial functioning of some children with UCBED should be noted by health insurance companies.

Parents appreciated the contact with other families with children with UCBED, probably because it brought recognition, emotional support, and appeared useful for practical advice [25]. Our results showed that not only parents [25], but also children and adolescents benefited from contact with similar others or felt the need to get in touch with similar peers.

### Comparing Perspectives

#### Children and adolescents

The negative feelings throughout early adolescence, which we detected in this study, are not necessarily related to UCBED and are also observed in able-bodied children [26]. Able-bodied children and adolescents also appear to go through different phases in life, and body image and psychological wellbeing seemed to be related most strongly during adolescence [27]–[30]. Despite the negative feelings associated with early adolescence, children and adolescents from the age of thirteen and older often stated that they no longer wanted a sound hand, in contrast with the younger children. This says something about the level of acceptance at different ages. Only one group of children (13–16 years) considered contact with similar others and support from the rehabilitation team very valuable, while these were not mentioned as helpful by the other age groups. Perhaps, it is typical for puberty to find these things to learn to cope with the short arm important. During the interviews, participants made some remarks about making contact and starting up relationships, but the subject sexuality has not been discussed during the study week. Nor did we explicitly ask about it. However, it is remarkable that sexuality was not mentioned at all, and further research into this topic would be interesting.

#### Prosthesis wearers and non-wearers

It seems that non-wearers were more resilient, used humour more often in response to reactions, and were more willing to provide explanations about the deficiency. However, non-wearers also tended to hide their arm more often than wearers. This seems to contradict the conclusion that they are more resilient than wearers. Maybe, non-wearers do not always feel like explaining their deficiency and then hiding the arm seems to be their strategy to resist the staring. Wearers do not need this hiding strategy, since their deficiency is not that visible through the prosthesis. For wearers, wearing a prosthesis could be of value in avoiding inconvenient environmental factors and can therefore help a child build up confidence and prevent from negative feelings caused by staring. These aspects of wearing a prosthesis should be incorporated more explicitly into the information provided to parents and children.

#### Parents and children

Parents reported fewer negative feelings than children did and thus overestimated the psychosocial functioning of their child. This was in contrast to Ylimäinen et al.’s findings [5] that parents tend to overemphasise the problems of a child with a deficiency. However, these results concerned health-related quality of life, which is not the same as psychosocial functioning. Our results may reflect the parents’ judgment of the functioning of their child as a whole. Functioning also contains other aspects, such as performing activities and participation [31], in addition to psychosocial elements. Parents could unfairly assume their children to be functioning well psychosocially, because they lack activity limitations or participation restrictions.

#### Parents

The parental emphasis on the importance of their child’s personality in learning to cope with UCBED was remarkable. They believed that special features in their child’s personality had contributed to the ability to cope so well with the deficiency. In previous research, parents also described their children with upper limb differences as strong, resourceful and intelligent individuals who coped very well with their deficiency [4]. These results indicate that there could be personality differences between children with UCBED and those without physical differences – raising the question whether having a visible deficiency is character forming.

#### Health professionals

The health professionals’ emphasis on the importance of support from parents of children with UCBED was striking. This finding confirmed previous investigations stating that more adaptive parental psychological adjustment was associated with positive psychological adjustment [32]. A study among able-bodied adolescents also proved that the family environment was the most important factor in explaining high levels of self-esteem despite poorer perceptions of personal appearance [26]. However, the parents in our study did not seem to be aware of their great influence. This could be a task for rehabilitation care; raising parental awareness of their influence on the psychosocial functioning of their children. Another difference in the perspectives of health professionals and the other participating groups was that professionals stressed the importance and possibilities of psychosocial help given by specialists in rehabilitation teams. During the online focus group interviews, however, children and adolescents indicated that they did not feel the need to talk to professionals about the deficiency. Contact with similar others can be regarded as an important form of psychosocial help and should be incorporated more structurally into rehabilitation care. In addition to peer-to-peer contact during specially organised days, social media can also play a part. Online discussion forums like the one we used in our study are a modern and easy way for children and adolescents with UCBED to get in touch with peers, and appeared to be a form which was greatly appreciated by participants. Furthermore, since support from parents is of great influence in how children with UCBED cope with the deficiency, it is also advisable to involve parents in psychosocial treatment. Previous investigation of children with cerebral palsy has already recommended interventions including family members, since the resilience and successful adaptation of parents appeared to be associated with effective coping in children [33].

### Strengths of the Study

Our study was the first to investigate psychosocial functioning from the children’s and adolescents’ perspectives. Furthermore, the online focus group interviews were held with several groups of participants, which made it possible to compare perspectives.

The response rates of participants who took part in the online focus group interviews varied from approximately fifty to eighty percent. These high response rates and the great number of remarks made on psychosocial functioning during the interviews enabled us to provide more insight into the psychosocial functioning of children and adolescents with UCBED. By holding the focus group interviews online, participants were completely anonymous. Anonymity could be very important, especially in research into the feelings of participants. This methodology could have contributed to participants being more open and less reserved in sharing their feelings, compared to a live focus group interview.

### Study Limitations

Participants were recruited at random from several rehabilitation centres and patient organisations, to ensure an appropriate reflection of the general population of children and adolescents with UCBED and their parents was obtained. Age, gender and referring centre or patient organisation were distributed equally across groups (Table 1). However, because of outdated information provided by rehabilitation centres, there was an unequal proportion of prosthesis wearers and non-wearers in the youngest child and parents group. The under-representation of prosthesis wearers in these two groups could have introduced some bias into the results, since fewer remarks could contribute to a less extensive view on psychosocial functioning. For the analysis, however, we combined the data from all wearers and non-wearers from the three child and adolescent groups. As a result, there were sufficient remarks from both wearers and non-wearers about psychosocial functioning to draw conclusions about the differences between the two.

### Conclusion

Children and adolescents with UCBED had mixed feelings about their deficiency. Both negative and positive feelings could be experienced simultaneously, and mainly depended on the way people in the environment reacted to the deficiency. Staring was the kind of reaction mentioned most frequently and affected the psychosocial functioning of children negatively. Support from people in the environment could help children with UCBED to cope with their deficiency. Other coping strategies were wearing a prosthesis and contact with peers with UCBED. Differences in the psychosocial functioning of prosthesis wearers and non-wearers showed that non-wearers tended to be more resilient and that wearers wore their prostheses mainly to avoid negative reactions from the environment and for cosmetic reasons. From our results, we advise the rehabilitation team to make parents more aware of the great influence they have on the psychosocial functioning of their children. And finally, we advise to make adjustments to the psychosocial help given by the rehabilitation team, since help in the form of conversations with psychologists appeared not to be helpful, and to encourage peer-to-peer contact.
